# Frequency of *EGFR* Mutations in 907 Lung Adenocarcioma Patients of Indian Ethnicity

**DOI:** 10.1371/journal.pone.0076164

**Published:** 2013-10-04

**Authors:** Anuradha Chougule, Kumar Prabhash, Vanita Noronha, Amit Joshi, Abhishek Thavamani, Pratik Chandrani, Pawan Upadhyay, Sagarika Utture, Saral Desai, Nirmala Jambhekar, Amit Dutt

**Affiliations:** 1 Department of Medical Oncology, Tata Memorial Hospital, Tata Memorial Center, Mumbai, Maharashtra, India; 2 Advanced Centre for Treatment, Research and Education in Cancer, Tata Memorial Center, Navi Mumbai, Maharashtra, India; 3 Department of Pathology, Tata Memorial Hospital, Tata Memorial Center, Mumbai, Maharashtra, India; Sanjay Gandhi Medical Institute, India

## Abstract

**Background:**

During the past decade, the incidence of *EGFR* mutation has been shown to vary across different ethnicities. It occurs at the rate of 10–15% in North Americans and Europeans, 19% in African-Americans, 20–30% in various East Asian series including Chinese, Koreans, and Japanese. Frequency of *EGFR* mutations in India however remains sparsely explored.

**Methodology/Principal Findings:**

We report 23% incidence of *Epidermal growth factor receptor* (*EGFR*) mutations in 907 Non small cell lung cancer (NSCLC) patients of Indian ethnicity, in contrast to 10–15% known in Caucasians and 27–62% among East Asians. In this study, *EGFR* mutations were found to be more common in never-smokers 29.4% as compared to smokers 15.3%. Consistent with other populations, mutation rates among adenocarcinoma-males were predominantly lower than females with 32% incidence. However unlike Caucasians, *EGFR* mutation rate among adenocarcinoma-never-smoker females were comparable to males suggesting lack of gender bias among never smokers likely to benefit from EGFR targeted therapy.

**Conclusions/Significance:**

This study has an overall implication for establishing relevance for routine *EGFR* mutation diagnostics for NSCLC patients in clinics and emphasizes effectiveness for adoption of EGFR inhibitors as the first line treatment among Indian population. The intermediate frequency of *EGFR* mutation among Indian population compared to Caucasians and East Asians is reminiscent of an ancestral admixture of genetic influence from Middle Easterners, Central Asians, and Europeans on modern- Indian population that may confer differential susceptibility to somatic mutations in *EGFR*.

## Introduction

In India, and worldwide, lung cancer is the leading cause of cancer mortality. About 63 thousand new cases are diagnosed each year in India with deaths estimated at approximately 52,000, accounting for about 8% of all cancer deaths [1], [2]. The majority of lung cancer cases are non-small cell lung cancers (NSCLC), which accounts for approximately 85% of all lung cancer cases. Of the available therapeutic options, chemotherapy for NSCLC has been only marginally effective. Development of new and effective therapy therefore has been and continues to be a major public health imperative. Among a multitude of targeted agents explored for the treatment of advanced NSCLC, small molecule tyrosine kinase inhibitors (TKI), gefitinib and erlotinib, targeting a mutant epidermal growth factor receptor (EGFR) have shown significant benefit in clinics. Activating *EGFR* mutations in the tyrosine kinase region have been shown to underlie response to these inhibitors, and have become an established predictive marker to select NSCLC patients for treatment [3]–[5]. FDA approved EGFR inhibitors as the first line of treatment for advanced NSCLC patients positive for *EGFR* activating mutation [6], [7], but not for patients with wild-type *EGFR*, wherein in contrast an inferior outcome is observed in response to the treatment [8], [9]. Thus, an accurate incidence of *EGFR* mutation findings is critical to reckon the effectiveness for adoption of gefitinib as first line of treatment for *EGFR*-activating mutation-positive advanced NSCLC patients in any given population.

During the past decade, the incidence of *EGFR* mutation in NSCLC has been shown to vary across different ethnicities. It occurs at the rate of 10–15% in North Americans and Europeans [3], [4], [10], 19% in African-Americans [11], 26–30% in various East Asian series including Chinese [12], Koreans [13], Japanese [14], and as compiled in Table 1 (adapted from [15]). Frequency of *EGFR* mutations in India however remains sparsely explored. Although, there are three reports from India with mutation rate varying between 22–51.8%, they tend to overestimate the incidence of *EGFR* mutation, because of a small sample size and clinically selected patients [16]–[18]. Here, in this study we determine *EGFR* mutation rate in 907 NSCLC patients of Indian ethnicity and correlate across different variables of age, gender, habits and histology groups.

**Table 1:** Molecular Epidemiological status of EGFR mutation (adapted [15]).

Region	Incidence	Overall Mutation Rate
Vietnam	77/120	64.2%
Japan	71/263	27%
East Asia	107/361	30%
Taiwan	108/174	62.1%
Thailand	63/117	53.8%
Philippines	34/65	52.3%
China	372/741	50.2%
Hong Kong	76/161	47.2%
United States of America	11/80	14%
Australia	6/83	7%
India - Chennai	16/72	22.2%
India (Tata MemorialHospital)	202/780	26%

## Materials and Methods

This study is a retrospective analysis on all the patients who were referred for *EFGR* testing from medical oncology department at the Tata Memorial Hospital, Mumbai as a routine service over a 1.5 year period. This was part of standard care: when referred for *EGFR* genotyping, the diagnosis of adenocarcinoma or squamous cell carcinomas were made on histomorphological grounds in cases where the appearances were characteristic, or immunohistochemistry staining were performed using antibodies against TTF1, P53, Napsin A, and CK 5/6. As a routine practice, 2 or more antibodies were used to distinguish adenocarcinoma from squamous carcinoma. Smoking history was recorded by directly asking specific question to all the patients at the Medical Oncology Department, Tata Memorial Hospital. The Institutional Review Board (IRB) and the Ethics Committee (EC) of Tata Memorial Center (TMC)- Advanced Centre for Treatment, Research and Education in Cancer (ACTREC) (Mumbai, India) approved the project (#108) during the 21st TMC-ACTREC IRB meeting. Since this was a retrospective analysis, the IRB and the EC waived the need for an informed consent. Patients were randomly selected based on the availability of biopsy block from the database maintained in the Medical Oncology Department at Tata Memorial Hospital. The patient characteristics including the age, gender, smoking/tobacco use and histopathology were recorded.

### Collection of Patient Samples

The paraffin embedded FFPE blocks of the patients were collected from the pathology department, at the Tata Memorial Hospital. The hematoxylin and eosin stained sections of the blocks were mounted on slides and viewed under the microscope to confirm that the tumor – region constitutes more than 75% of the tissue mass.

### DNA Extraction

For DNA isolation, six FFPE tissue sections of 14 µm each were taken, using microtome (Leica). After deparaffinizing the sections with Xylene, the DNA was extracted as per the kit insert (QiaAmp FFPE tissue kit, Cat no 56404) and as described earlier [19].

### Mutation Analysis by PCR and Sequencing

Extracted DNA was amplified for the exons 18, 19, 20 and 21 using a nested-PCR method [20], with a 100 ng DNA as the template. With the above PCR products as template, second round of nested PCR amplification was carried out using a different set of primers flanking the regions. The amplicons were then purified using the Qiaquick PCR purification kit (Qiagen). About 2.5 ng of the PCR product, along with 1.6 pmols of the forward or reverse primer was used for sequencing in the Applied Biosystems DNA Analyser, as described earlier [21]. The resulting sequence was compared to the available normal sequence in the NCBI database using the NCBI BLAST and the variations observed thereupon were recorded.

### Mutation Analysis by TaqMan based Real Time PCR Technique

The reaction mixture was carried out in 10 µL volumes, which contains 5 µL of the Taqman probe, master mix (Roche), primers at a final concentration of 9 µM and probes at a final concentration of 2 µM and the remaining volume was made up to 5 µL using PCR grade water using LC 480 II machine. The program used was, 50°C for 2 minutes and 95°C for 10 minutes, followed by 40 cycles of 95°C for 15 seconds and 60°C for 1 minute. The detectors used were FAM/VIC at 5′ and TAMRA/MGB at 3′ fluorescence for the wild type and mutant respectively. The Assay sensitivity has a limit of 1% detection and each run consisted of a positive and a negative control to check the integrity of the assay.

### Statistical Analysis

The Chi-square test was performed to reveal any significant correlation between the mutation status and age, gender and habits of the patient and tumor histology.

## Results

### Clinical Testing for *EGFR* Exon 18–20 in 907 Consecutive Cases

**Figure 1:**
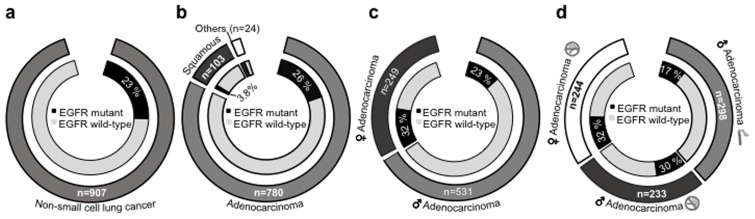
*EGFR* (exon 18–21) mutation status among NSCLC patients in India. (A) *EGFR* mutation status across all NSCLC samples is shown. Black segment in the inner circle indicate patients harboring *EGFR* mutation, the segment in white indicates patients with wild type *EGFR*. The segment in grey (outer circle) indicates total number of NSCLC patients. (B) *EGFR* mutations across different histological subtypes. Grey segment in the outer circle indicate total number of lung adenocarcinoma patients. The patients with squamous histology are represented in dark grey. The segment in white represents lung cancer patients with tumors histology other than adeno or squamous carcinoma. Similar to (A), the black segment in the inner circle indicate patients harboring *EGFR* mutation, the segment in white indicates patients with wild type *EGFR*.(C) *EGFR* mutation variation among male and female lung adenocarcinoma patients. The light grey slice represents male patients; female lung adenocarcinoma patients represented in dark grey in outer circle. Similar to (A), the black segment in the inner circle indicate patients harboring *EGFR* mutation, the segment in white indicates patients with wild type *EGFR* among male and female lung adenocarcinoma patients. (D) *EGFR* mutation variation with respect to gender specific smoking habits among all lung adenocarcinoma patients. Light grey segment in the outer circle indicate male adenocarcinoma lung cancer patients with smoking history. The non smoker male and female lung adenocarcinoma patients are represented in dark grey and white, respectively. The segment in white represents lung cancer patients with tumors histology other than adeno or squamous carcinoma.

907 patients diagnosed with lung cancer in Tata Memorial Hospital from August 2011 to December 2012 were tested for common *EGFR* mutation subtypes by real time PCR using TaqMan primer probes for point mutations in exon 18 and 21, and an in frame deletion in exon 19. Exon 20 point mutation assay was incorporated from August 2012 onwards in 215 cases. After standardization and validation by sanger based directed sequencing for the robustness and sensitivity of the real time PCR, it was implemented as the routine diagnostic test (data not shown). As shown in Table 2, of the 907 patients tested, there were 642 males and 265 females with a ratio of 2.4∶1. The median ages of male and female population’s were 57 and 54 years respectively. No data of smoking habit was available in 31 males. In the group of <40 years, there were only 12 male smokers out of 92. Within the different age group the mutation rate was marginally high in >60 yrs as compared to age of ≤60 yrs (26.8% vs 21). The total population of smoker vs never-smoker was 39.6% vs 56.8% with a p value of <0.001 (Table 3). Overall mutation rate was 23.2% with a higher mutation rate in females as compared to males (29.8% vs 20%) with a p value of 0.002 (Table 3), and as shown in Fig. 1.

**Table 2:** Patient Demographics.

N = 907	Variables	Smokers	Non –smokers	NA
No of Patients		360 (39.6%)	516 (56.8%)	31(3.4%)
Mean Age		58.14	52.9	54.7
Age	<40 yrs	12	76	4
	40–60 yrs	180	277	18
	>60 yrs	168	163	9
Gender	Male	353	258	31
	Female	7	258	0
Histopathology	Adenocarcinoma	277	477	26
	Squamous	74	26	3
	Adenosquamous	5	6	2
	Others	4	7	0

**Table 3:** Frequency and Overall mutation rate of different variables in NSCLC.

Variables	Mutations	Total	Percentage	Significance
**Over all Mutation Rate**	210	907	23.0%	0.002
**Male**	131	642	20.4%	
**Female**	79	265	29.8%	
**Smoking status**				
**Smokers**	55	360	15.3%	<0.001
**Never smokers**	152	516	29.4%	
**Not available**	3	31	9.7%	
**Histopathology**				
**Adenocarcinomas**	202	780	25.9%	<0.001
**Squamous**	4	103	3.8%	
**Others**	4	24	16.7%	
**Gender with histopathology**				
**Male adenocarcinomas**	123	531	23.0%	0.01
**Female adenocarcinoma**	79	249	32.0%	
**Gender with smoking status**				
**Never smoker male**	73	258	28.3%	0.56
**Never smoker female**	79	258	30.6%	
**Histopathology with smoking status and gender**				
**Adenocarcinomas never smoker male**	70	233	30.0%	0.58
**Adenocarcinomas never smoker female**	79	244	32.0%	

### Association of *EGFR* Mutations with the Smoking Status of the Patients

There were equal numbers of never-smoker in both genders i.e. 258, this number in both the groups allowed us to adequately compare the two groups. *EGFR* mutation rate was more prevalent in the never smoker than the smoker group (29.4% vs 15.3%) with a p value of <0.001 (Table 3; and Fig. 1). However, it was almost equivalent in the never-smoker group of males and females (28.3%vs. 30.6%), as shown in Fig. 1.

### Association between *EGFR* Mutation and Clinicopathological Status of the Patients

Majority of the histological diagnoses examined were adenocarcinoma (88.0%), followed by squamous cell carcinomas (11.6%) and adenosquamous carcinomas (1.5%) (Table 2). The overall frequency of *EGFR* mutations in adenocarcinoma population was 26% as compared to 3.8% in squamous cell carcinomas (Table 3; and Fig. 1). Interestingly, mutation rate was predominantly higher among women than male adenocarcinomas (32% vs 23%) but there was no significant difference between never-smoker female and male (32% vs 30%) adenocarcinomas. Of all the *EGFR* TK domain mutations 50% were in-frame deletions in exon 19, while 42% were missense mutations in exon 21, 7% of the mutations are reported in exon 18 and 3% in exon 20 with two patients harboring mutation in exon 20 along with exon 21.

## Discussion

Many countries have already adopted in practice testing for *EGFR* mutations to determine appropriate treatment with EGFR inhibitors. While there are still some uncertainties whether the superior outcome observed in the clinical trials are generalizable across different ethnicities, different countries continue to evaluate clinical utility and cost-effectiveness of these inhibitors. We recently showed 74% response rate to oral tyrosine kinase inhibitor among Indian NSCLC patients with activating mutation of *EGFR* compared to 5% among patients with wild type *EGFR*, emphasizing *EGFR* mutation as an important predictive marker for response to oral tyrosine kinase inhibitors in a clinically selected small cohort of 111 patients of Indian origin [16]. It is thus imperative to know the mutation rates of this biomarker accurately by involving larger unbiased sample size to allow adequate statistical power for analysis. This study of 907 patients (largest single cohort study from India) showed an overall 23% *EGFR* mutation, with a rate of 26% in adenocarcinoma, in Indian population that closely approximates the rate in Japanese and East Asian countries. We found a higher rate of *EGFR* mutation in adenocarcinomas than over all (29.8% vs 20%) and among women than men (32% vs 23%), similar to Korean, Chinese and Japanese data as reported earlier [1], [13]–[15] (Table 1). *EGFR* mutations have been consistently reported to be more common in never-smokers as compared to smokers [11], which our study confirms as well (29.4% vs 15.3%). Though, the mutation rate varies significantly between never-smokers and smokers across different ethnicities. For e.g., 47% never smokers harbor *EGFR* mutations compared to 22% among smokers in the Chinese population; among other Asian series, mutation rate among never-smokers have been reported to be as high as 60%; among European descent, 22% Italians and 38% Spanish never-smokers series were reported to be *EGFR* mutation positive compared to 8% smokers [11], [22]. Histopathologically, mutation rates among adenocarcinoma-males were predominantly lower than females, consistent with literature. However, no significant difference were observed between never-smoker adenocarcinoma females and males indicating lack of gender bias among never-smoker patients, possibly due to lower proportion of male non smokers (258 non smokers out of 516 males) exists in India compared to female non smokers (258 non smokers out of 265 females), unlike in developed countries. This is also consistent with similar studies on never-smoker females of East Asian ethnicity [23]–[25], wherein rate of mutation varies with clinical stage of never-smoker female patients [26]. Among the squamous cell lung carcinomas, there have been few studies within East Asians with conflicting results: two study from China reported *EGFR* mutation rate of 21% (3/14) and 13.3% (4/30) compared to a Korean based study with 7.3% (3/41) [27]–[29]. We found *EGFR* mutation rate of 3.8% in this largest cohort of squamous cell carcinoma study reported so far, n = 103, which is surprisingly more similar to incidence as observed in the west. Taken together, this study emphasizes that *EGFR* mutation test for squamous cell carcinoma need to as well be considered as a routine practice, as recommended for *ALK* mutations with comparable incidence at around 5% [30].

Our findings of exon 18, 19 and 21 with a mutation rate of 7%, 50% and 42% respectively is similar to that described in Spanish and Japanese populations [22], [31]–[35]. In our earlier study, exon 19 mutation was present in 67% cases and exon 21 in 29% of cases, this difference could be because of the smaller sample size of 111 patients [16]. Within the cohort of different mutations, we analyzed the clinical and pathological characteristics of lung adenocarcinoma patients. Our results suggest that exon 21 mutations are more common in never- smoker females than males, whereas exon 19 showed marginally higher mutation rate in never smoker males than females. It has been reported that exon 18 *EGFR* mutations are detected more frequently in younger patients, which was also noted in our study [32]. Of note, some other studies have also observed no association between *EGFR* mutations and age, gender or smoking history in Chinese patients [23]. Furthermore, the analysis involving exon 20 mutations, performed in 215 out 907 patients, identify 3% incidence similar to the reported literature [35].

A major limitation of this study is the lack of survival data. Since the cases were collected from 2011 to 2012, survival data of these patients were far from maturity. While we recently reported clinical correlation to *EGFR* mutation status on a retrospectively collected smaller independent cohort of 111 patients, it would be of interest to validate the prognostic impact of clinical variables, histologic patterns, and mutation types on these 907 patients. In summary, western series show a mutation rate of 10–15% [4], [10] and East Asian population show a heterogeneous mutation rate of 27–62% [22], [34], [35]. Mutation rate of 23% in *EGFR* among Indian population is less than that of East Asian patients and more than Western patients. This finding is interestingly reminiscent of the historical evidence for amalgamation of major ancestral populations over past thousand of years in India creating an admixture of genetic influence from Middle Easterners, Central Asians, and Europeans due to different degree of migration wave at different points of time in the Indian history [36]. Furthermore, this study could plug the crucial deficit in establishing gradient incidence of *EGFR* mutation across ethnicities and help inform designing of genome wide association studies to determine haplotype that may confer differential susceptibility to somatic mutations in *EGFR* in NSCLC, as reported for somatic mutations in *JAK2* in human myeloproliferative neoplasms [37].
